# Influences of Product Temperature on Emotional Responses to, and Sensory Attributes of, Coffee and Green Tea Beverages

**DOI:** 10.3389/fpsyg.2017.02264

**Published:** 2018-01-11

**Authors:** Ragita C. Pramudya, Han-Seok Seo

**Affiliations:** Department of Food Science, University of Arkansas, Fayetteville, AR, United States

**Keywords:** product temperature, sensory attribute, emotional response, coffee, green tea, gender

## Abstract

Coffee and green tea are popular beverages consumed at both hot and cold temperatures. When people consume hot beverages concurrently with other activities, they may experience at different temperatures over the period of consumption. However, there has been limited research investigating the effects of product temperatures on emotional responses and sensory attributes of beverages. This study aimed to determine whether emotional responses to, and sensory attributes of, brewed coffee and green tea vary as a function of sample temperature. Using a check-all-that-apply (CATA) method, 157 participants (79 for coffee and 78 for green tea) were asked to evaluate either coffee or green tea samples served at cold (5°C), ambient (25°C), and hot (65°C) temperatures with respect to emotional responses and sensory attributes. The results showed that sample temperature could have significant influences on emotional responses to, and sensory attributes of, coffee and green tea samples. More specifically, 6 and 18 sensory attributes of coffee and green tea samples, respectively, significantly differed with sample temperature. Beverage samples evaluated at 65°C were characterized, regardless of activation/arousal level, by positive emotional responses terms and favorable sensory attributes. While beverages evaluated at 25°C were associated more with negative emotional responses with low activation/arousal, those evaluated at 5°C were more frequently characterized as having negative emotional responses with high activation/arousal. Sensory and emotional drivers of liking for both coffee and green tea differed both with sample temperature and gender. While both emotional responses and sensory attributes were identified as drivers of liking among females, only emotional responses were identified as drivers of liking among males. In conclusion, this study provides empirical evidence that both emotional responses to, and sensory attributes of, coffee and green tea beverages can vary with sample temperatures. To provide a better understanding of product characteristics, emotional responses to, and sensory attributes of, coffee or green tea beverages should be tested over a wider range of product temperatures.

## Introduction

Serving temperatures have been found to influence perceived intensities in basic taste solutions (Moskowitz, 1973; Bartoshuk et al., 1982; Lipscomb et al., 2016). Moreover, serving temperatures have been found to affect flavor/taste intensities and acceptances of various beverage products, including milk (Francis et al., 2005), wine (Zellner et al., 1988; Ross and Weller, 2008; Cliff and King, 2009), carbonated beverages (Cardello and Maller, 1982), and fruit-flavored beverages (Zellner et al., 1988). Those earlier studies, however, focused on quantification of intensity variation rather than qualification of sensory attributes. In other words, limited research has been done to examine whether detectability of certain sensory attributes can be affected by serving temperature of food or beverage products.

There has been no research regarding the effects of product temperatures on emotional responses to food or beverage products. Research investigating how product temperatures affect associations between emotional response and sensory perception of food or beverage products is also limited. However, three points are worth noting. First, food-evoked emotions play an important role in food acceptance and choice (King and Meiselman, 2010; Dalenberg et al., 2014; Gutjar et al., 2015). Furthermore, measuring both evoked emotions and sensory perception has been found to yield better understanding of consumer acceptance and preference toward foods or beverages (Samant et al., 2017). Second, thermal sensation (physical warmth or coldness) has been found to evoke emotional responses in humans (Kanosue et al., 2002; Sung et al., 2007; Williams and Bargh, 2008). Neuroimaging studies have revealed that when the body is exposed to different temperatures, significant changes of neural activations can be observed in the brain regions responsible for emotion processing, as well as thermal sensory perception (Kanosue et al., 2002; Sung et al., 2007; Rolls et al., 2008; Rolls, 2010). In a functional magnetic resonance imaging study conducted by Guest et al. (2007), liquid stimuli into the mouth at three different temperatures (5, 20, and 50°C) increased neural activation in the brain regions associated with taste perception and reward, such as the insula, the somatosensory cortex, the orbitofrontal cortex, the anterior cingulate cortex, and the ventral striatum. In particular, pleasantness ratings of oral thermal stimuli were correlated with neural activations in the orbitofrontal cortex and the pregenual cingulate cortex. Finally, when people consume hot or cold meals concurrently with other activities like engaging in social conversations or performing office work, they may experience their meals over a wider range of food or beverage product temperatures because the temperatures decrease with time (Pramudya and Seo, 2018); it was reported that people generally consume a meal over a time interval between 10 and 60 min (Bell and Pliner, 2003).

This study aimed to determine whether and how temperatures of product samples affect emotional responses to, and sensory attributes of, brewed coffee and green tea beverages consumed at different temperatures: hot (65°C), ambient (25°C), and cold (5°C) temperatures. These three values were chosen because those typically encounter during consumption of coffee and green tea beverages in daily life. More specifically, brewed coffee and green tea beverages are often consumed at hot temperatures; university students in the United States rated the range of 62.8–68.3°C as ideal for consuming coffee beverages (Borchgrevinka et al., 1999). In addition, when people consume hot beverages while engaged in other activities (e.g., social conversation or office work) over a period of time, initially hot beverage temperature may fall to near ambient temperature (25°C) during consumption. Finally, coffee and green tea continue to gain popularity as cold beverages (5°C), e.g., iced coffee and iced matcha. Coffee and green tea beverages were specifically chosen as target products for this study because both are widely popular beverages consumed across numerous cultures worldwide, and are considered as “emotional” beverages that provide psychological comfort (Juneja, et al., 1999; Cooper, 2012; Bhumiratana et al., 2014; Labbe et al., 2015).

Four research propositions were tested in this study. First, it was to be determined whether specific sensory attributes of coffee or green tea samples would be more detectable or dominant at hot, ambient, or cold temperatures (Research proposition 1), based on previous research regarding the effects of serving temperatures on intensities of sensory attributes in basic taste solutions, foods, and beverages (Moskowitz, 1973; Zellner et al., 1988; Ross and Weller, 2008; Kim et al., 2015; Lipscomb et al., 2016; Stokes et al., 2016; Steen et al., 2017). Due to temperature-dependent variations with respect to perceived intensity, certain attributes may be more dominant in coffee or green tea samples at hot, ambient, or cold temperature. Steen et al. (2017) evaluated brewed coffee samples at six serving temperatures ranging from 62 to 31°C by measuring volatile compound profiles using gas chromatography-mass spectrometry and eight flavor attributes (overall intensity, sour, bitter, sweet, tobacco, roasted, nutty, and chocolate) using descriptive sensory analysis. Intensities of four sensory attributes, i.e., overall intensity, bitter note, sweet note, and roasted flavor, were found to differ with sample temperatures. These attributes were especially associated with brewed coffee samples evaluated at temperatures of 50°C or higher, possibly due to greater levels of aliphatic ketones, alkylpyrazines, some furans, and pyridines (Steen et al., 2017).

Second, it was to be determined whether specific emotions would be more highly evoked at hot, ambient, or cold temperature of coffee or green tea samples (Research proposition 2). Since sensory attributes have been found to be associated with emotional responses, temperature-induced variations in sensory attributes might be expected to affect emotional responses toward coffee or green tea beverage samples served at hot, ambient, and cold temperatures (Seo et al., 2009c; Ng et al., 2013; Chaya et al., 2015). Prior research has also demonstrated that warm or cold stimuli to the whole or partial body (e.g., hands or legs) can affect not only hedonic valence, but also emotional responses such as thermal comfort or discomfort (Kanosue et al., 2002; Guest et al., 2007; Sung et al., 2007; Rolls et al., 2008).

Third, based on previous research that found gender differences with respect to sensory perception (Doty et al., 1984; Larsson et al., 2003; Royet et al., 2003; Doty and Cameron, 2009; Ferdenzi and Roberts, 2013) and emotional processing (Wager et al., 2003; Seo et al., 2009c; Duerden et al., 2013), it was to be determined whether the effects of sample temperatures on sensory attributes and emotional responses would vary with gender (Research proposition 3). Females have been found to show better performances than males in odor memory, odor identification, and verbal fluency tasks (Larsson et al., 2003), possibly because of the greater number of neural activations of the left orbitofrontal cortex in females (Royet et al., 2003). Behavioral and neuroimaging studies have also demonstrated that males are more attentive to sensory aspects of emotional stimuli, while females are more attentive to subjective feelings of emotional stimuli (Orozco and Ehlers, 1998; Wager et al., 2003). A recent meta-analysis of neuroimaging studies associated with gender differences in emotional processing found that female processing of emotional stimuli occurs predominantly in the bilateral anterior insula as well as the mid and posterior insula on the left side, while males respond to emotional stimuli predominantly in the left anterior and mid insula as well as in the right posterior insula (Duerden et al., 2013). For this reason, it was anticipated that product temperature-dependent variation with respect to sensory attributes and emotional responses would be more pronounced in females than in males.

Finally, both sensory attributes and emotional responses have been found to play an important role in consumer acceptance of food or beverage products (Seo et al., 2009c; King and Meiselman, 2010; Piqueras-Fiszman and Spence, 2012; Dalenberg et al., 2014; Gutjar et al., 2015; Samant et al., 2017). It was therefore to be determined whether the impacts of sensory attributes and emotional responses on liking of coffee or green tea beverages would vary as a function of sample temperature (Research proposition 4a) and gender (Research proposition 4b). More specifically, if specific sensory attributes (Research proposition 1) and/or emotions (Research proposition 2) would be predominantly present at hot, ambient, or cold temperatures of coffee or green tea samples, the relative impact of individual sensory and emotional responses on liking of those samples may differ as a function of sample temperature. Moreover, if product temperature-induced sensory attributes and/or emotions differ by gender (Research proposition 3), the relative impacts of individual sensory and emotional responses on liking of coffee or green tea samples may differ between females and males.

The present study was designed to test the four research propositions for coffee (Study 1) and green tea (Study 2) beverages. This study was conducted in conformance with the Declaration of Helsinki for studies on human subjects. The protocol used in this study was approved by the Institutional Review Board of the University of Arkansas (Fayetteville, AR, United States). A written informed consent was obtained from each participant prior to the participation.

## Study 1: Effects of Sample Temperatures on Emotional Responses to, and Sensory Attributes of, Coffee Beverage

### Materials and Methods

#### Participants

Through the consumer profile database of the University of Arkansas Sensory Service Center (Fayetteville, AR, United States), 79 coffee consumers (51 females and 28 males) ranging in age from 19 to 76 years [mean ± standard deviation (SD) = 39 ± 16] were recruited. Using a pre-screening survey, all participants self-reported that they habitually drink one or more cups of coffee with no added condiments, e.g., sugar, milk, and creamer, etc., and they prefer black coffee [i.e., greater than 5-points on a 9-point hedonic scale ranging from 1 (dislike extremely) to 9 (like extremely)]. Participants also self-rated preferences for hot beverages (mean ± SD = 7.9 ± 1.0) on a 9-point hedonic scale ranging from 1 (dislike extremely) to 9 (like extremely) and cold beverages (mean ± SD = 7.8 ± 1.3). All participants were asked to refrain from eating, drinking (except water), and cigarette smoking for 2 h prior to their participation to avoid potential influences of such activities on sensory perception (Cho et al., 2017).

#### Sample Preparation and Presentation

Grounded roasted coffee beans (Sugar Skull blend, Onyx Coffee Lab, Fayetteville, AR, United States) were brewed for 20 min using commercial coffee makers (Model DCC-2900, Cuisinart, East Windsor, NJ, United States) using a proportion of 90 g of ground coffee per 1,800-mL of spring water. A warm-up coffee sample (Lidl Essentials Coffee Classic, Lidl, Arlington, VA, United States) was prepared in the same manner. Brewed coffee was poured into a 3,000-mL stainless steel dispenser (Bunn, Springfield, IL, United States) to maintain its high temperature. Brewed coffee was served at three different temperatures: 65, 25, and 5°C. Sample preparation to achieve temperatures of 25 and 5°C involved placing coffee samples in a water bath to facilitate the cooling process. Each sample (55-mL) was presented in a 118-mL white Styrofoam cup identified with a three-digit code. Styrofoam cups were used to (1) minimize exposure of hands to thermal stimulation and (2) maintain target temperatures of coffee samples, and because Styrofoam cups are commonly used for serving both hot and cold beverages in the United States.

#### CATA Questions of Emotion and Sensory Tests for Coffee Beverage

Since temperatures of brewed coffee samples can change quickly over time, rapid methods of emotion and sensory testing were used in this study. More specifically, participants were asked to check all appropriate terms, listed on either emotion check-all-that-apply (CATA) question or sensory CATA question. This method was found to be suitable for characterizing product temperature-dependent sensory-attribute variations in foods and beverages (Chapko and Seo, 2017; Pramudya and Seo, 2018). The emotion CATA question included 39 emotion terms from the EsSense Profile^®^ (King and Meiselman, 2010). The sensory CATA question included 49 sensory attribute terms of coffee beverages generated by a previous study (Chapko and Seo, 2017). The following attributes were included: 21 aroma attributes (ashy, berry, bitter, brown sugar, burnt, cereal, chemical, chocolate, cocoa, fruity, green/vegetative, metallic, musty/earthy, nutty, papery/cardboard, pungent, roasted, skunky, sour, sweet, and tobacco); three appearance attributes (cloudy, oily, and transparent); 22 taste/flavor attributes (ashy, berry, brown sugar, burnt, cereal, chemical, chocolate, cocoa, fruity, green/vegetative, metallic, musty/earthy, nutty, papery/cardboard, pungent, roasted, skunky, tobacco, bitter taste, salty taste, sour taste, and sweet taste); and three mouthfeel attributes (astringent, mouth coating, and viscous). For each sensory modality (i.e., aroma, appearance, flavor, taste, and mouthfeel), the terms were presented in alphabetical order to assist participants in quickly finding all attributes that they wanted to check. Lee et al. (2013) showed that consumer panelists took significantly less time to answer CATA questions when the terms were listed in a fixed order rather than in the Williams design presentation order. It was also found that the influence of CATA term order on consumer responses was minimal (Lee et al., 2013).

#### Procedure

This study was conducted at the University of Arkansas Sensory Service Center (Fayetteville, AR, United States). Prior to sample presentation, each participant was given a verbal introduction to the experimental protocol. Participants were then asked to taste brewed coffee (not that used in actual testing) as a warm-up sample and select all appropriate terms from those listed on the emotion CATA question that characterized their emotional responses evoked by experiencing the sample (Varela and Ares, 2012). The warm-up session allowed participants to not only better understand both protocol and emotion CATA question, but also to minimize any carry-over effect.

Following the warm-up session, participants were asked to taste coffee samples at three different temperatures, i.e., 65, 25, and 5°C in a monadic sequential fashion. Participants were asked to drink each sample as much as they wanted, then select (as in the warm-up session) all the terms on the emotion CATA question for characterizing their emotional responses to the sample. The presentation order of the three serving temperatures was randomized over a time interval of 5 min. Following the evaluation of the three samples with respect to emotional response, participants were given a 5-min break prior to a sensory testing session. During each break, spring water (Clear Mountain Spring Water, Taylor Distributing, Heber Springs, AR, United States) and unsalted crackers (Nabisco Premium Unsalted Tops Saltine Crackers, Mondelez Global LLC, East Hanover, NJ, United States) were provided as palate cleansers.

Prior to the main sensory testing session, participants were asked to taste and evaluate with respect to sensory attribute a warm-up sample of brewed coffee. They were asked to select all sensory terms listed on the sensory CATA question for characterizing sensory attributes of the sample. Participants were then asked to taste and evaluate coffee samples at the same three temperatures used in the emotion testing session. Participants were also asked to provide their overall liking of each sample on a 9-point hedonic scale ranging from 1 (dislike extremely) to 9 (like extremely).

#### Statistical Analysis

Data were analyzed using XLSTAT statistical software (Addinsoft, New York, NY, United States) and SPSS 24.0 for Windows^TM^ (IBM SPSS Inc., Chicago, IL, United States). As previously proposed by Meyners et al. (2013) for an overall test of CATA data, chi-square testing was performed to determine whether the proportion of selections by participants for all terms of either the emotion CATA question or the sensory CATA question differed as a function of sample temperature or gender. To measure an effect size (or strength of association between two nominal variables) for chi-square test (or contingency table), Cramér’s *V* value was used. Cramér’s *V* values, ranging from 0 (no association between the variables) to 1 (perfect association), of 0.1, 0.3, and 0.5 were considered small, medium, and large effect-sizes, respectively (Cohen, 1988; Kittler et al., 2007).

Cochran’s *Q*-test (Cochran, 1950), using the exact probability and distribution of the *Q* statistic (Patil, 1975), was also performed to determine whether the proportions of selection by participants for individual terms of either the emotion CATA question or the sensory CATA question differed by sample temperature or gender. If significant differences were found among the variables, *post hoc* multiple pairwise comparisons were performed using the Marascuilo procedure (Marascuilo and McSweeney, 1967). Correspondence analysis, based on chi-square distance, was used to visualize relationships of sample temperatures to emotional responses and sensory attributes. Significant terms of the CATA questions, as determined by the Cochran’s *Q*-test, were used for correspondence analysis.

A three-way analysis of variance (ANOVA) was performed treating “sample temperature” and “gender” as main effects and “participant” as a random effect. If a significant difference in means was indicated by the ANOVA, *post hoc* comparisons between independent variables were performed using Tukey’s honest significant difference method. To measure an effect size for ANOVA, a partial eta squared ($\eta_{p}^{2}$) value was used; the $\eta_{p}^{2}$ values of 0.01, 0.06, and 0.14 are considered small, medium, and large effect-sizes, respectively (Kittler et al., 2007; Velasco et al., 2014). Penalty-lift analysis (Williams et al., 2011; Meyners et al., 2013) was also conducted to identify positive and negative drivers of overall liking among emotion and sensory attribute terms of coffee samples. Mean differences in overall liking between the selected and unselected cases for individual emotions and sensory attributes were then determined. A positive (or negative) value for a particular attribute indicates the mean liking of participants who selected that attribute was greater than the mean liking of those who did not (Meyners et al., 2013). A statistically significant difference was defined to exist when *P* < 0.05.

### Results

#### Overall Effects of Sample Temperatures on Emotional Responses and Sensory Attributes

To determine whether the proportions of selection by participants for all terms of either the emotion CATA question or the sensory CATA question differed as a function of sample temperature, the data were collapsed into the three temperature conditions: 5, 25, and 65°C. Chi-square testing revealed that the proportions of selection by participants for all emotion terms significantly differed among the three temperatures evaluated in this study (χ^2^ = 65.24, *P* < 0.001, *V* = 0.08): 5°C (12.3%), 25°C (12.7%), and 65°C (18.8%). More specifically, participants selected greater numbers of emotion terms when they evaluated coffee samples at 65°C than at 5 or 25°C, but the effect size (Cramér’s *V* value) was low. In addition, the selection proportions for all sensory terms were not significantly different among the three temperature conditions (*P* = 0.91): 5°C (18.6%), 25°C (18.4%), and 65°C (18.8%).

**Table 1** is a contingency table showing the proportions of selection by participants for individual emotion terms of coffee samples served at 5, 25, and 65°C. A higher proportion, i.e., closer to 1.00, indicates that the term was more frequently chosen by participants. Cochran’s *Q*-test revealed that 16 emotion terms of coffee samples significantly differed as a function of sample temperature: “active,” “bored,” “calm,” “disgusted,” “eager,” “energetic,” “glad,” “good,” “happy,” “nostalgic,” “peaceful,” “pleasant,” “pleased,” “satisfied,” “warm,” and “wild.” In addition, **Table 2** is a contingency table showing the proportions of selection for individual sensory-attribute terms of coffee samples served at the three temperatures. Cochran’s *Q*-test revealed that six sensory attributes of coffee samples significantly differed with respect to sample temperature: “pungent aroma,” “roasted aroma,” “metallic flavor,” “roasted flavor,” “skunky flavor,” and “bitter taste.”

**Table 1:** A contingency table of the proportions of selection by 79 participants for individual emotion terms among coffee samples evaluated at the three different temperatures.

Terms^1^	Sample temperatures			*Q*-value	*P*-value	Cramér’s *V* value
	5°C	25°C	65°C			
Active	0.25a	0.08b	0.24a	10.46	0.006	0.21
Bored	0.15ab	0.24a	0.09b	6.41	0.02	0.17
Calm	0.13b	0.25ab	0.33a	9.33	0.009	0.20
Disgusted	0.43a	0.39a	0.06b	28.26	<0.001	0.36
Eager	0.14ab	0.10b	0.25a	7.31	0.03	0.17
Energetic	0.29a	0.13b	0.28ab	7.48	0.02	0.18
Glad	0.10ab	0.05b	0.23a	10.76	0.004	0.22
Good	0.09b	0.18ab	0.33a	15.39	<0.001	0.25
Happy	0.14b	0.11b	0.33a	13.63	<0.001	0.24
Nostalgic	0.04b	0.09b	0.17a	7.60	0.02	0.18
Peaceful	0.09b	0.17b	0.33a	14.90	<0.001	0.25
Pleasant	0.19b	0.23ab	0.37a	7.24	0.03	0.17
Pleased	0.15b	0.17b	0.46a	23.53	<0.001	0.32
Satisfied	0.19b	0.10b	0.38a	16.84	<0.001	0.28
Warm	0.04b	0.04b	0.54a	68.09	<0.001	0.59
Wild	0.15a	0.14a	0.03b	8.67	0.01	0.19

*Cochran’s Q-test (Cochran, 1950), using the exact probability and distribution of the Q statistic (Patil, 1975), was performed to determine whether the proportions of participant selection for individual terms of emotion check-all-that-apply (CATA) question could differ by sample temperature. The proportions with different letters within each row represent a significant difference determined by post hoc multiple pairwise comparisons using the Marascuilo procedure (Marascuilo and McSweeney, 1967). Cramér’s V value was used to measure strength of association between two nominal variables (sample temperature × selected/unselected case) for the contingency table. Cramér’s V values, ranging from 0 (no association between the variables) to 1 (perfect association), of 0.1, 0.3, and 0.5 were considered small, medium, and large associations, respectively (Cohen, 1988). ^1^Only significant terms, determined by Cochran’s Q-test, among 39 emotion terms of the EsSense Profile^®^ (King and Meiselman, 2010) were shown (P < 0.05).*

**Table 2:** A contingency table of the proportions of selection by 79 participants for individual sensory attribute terms among coffee samples evaluated at the three different temperatures.

Terms^1^	Sample temperatures			*Q*-value	*P*-value	Cramér’s *V* value
	5°C	25°C	65°C			
**Aroma**						
Pungent	0.19a	0.06b	0.14ab	6.08	0.049	0.15
Roasted	0.35b	0.52ab	0.54a	6.86	0.03	0.17
**Taste/flavor**						
Metallic	0.33a	0.22ab	0.15b	7.74	0.02	0.17
Roasted	0.33b	0.37b	0.63a	18.00	<0.001	0.27
Skunky	0.19a	0.06b	0.08b	9.58	0.01	0.18
Bitter taste	0.84a	0.84a	0.67b	8.05	0.02	0.19

*Cochran’s Q-test (Cochran, 1950), using the exact probability and distribution of the Q statistic (Patil, 1975), was performed to determine whether the proportions of participant selection for individual terms of sensory check-all-that-apply (CATA) question could differ by sample temperature. The proportions with different letters within each row represent a significant difference determined by post hoc multiple pairwise comparisons using the Marascuilo procedure (Marascuilo and McSweeney, 1967). Cramér’s V value was used to measure strength of association between two nominal variables (sample temperature × selected/unselected case) for the contingency table. Cramér’s V values, ranging from 0 (no association between the variables) to 1 (perfect association), of 0.1, 0.3, and 0.5 were considered small, medium, and large associations, respectively (Cohen, 1988). ^1^Only significant terms, determined by Cochran’s Q-test, among 49 sensory attribute terms were shown (P < 0.05).*

**FIGURE 1:**
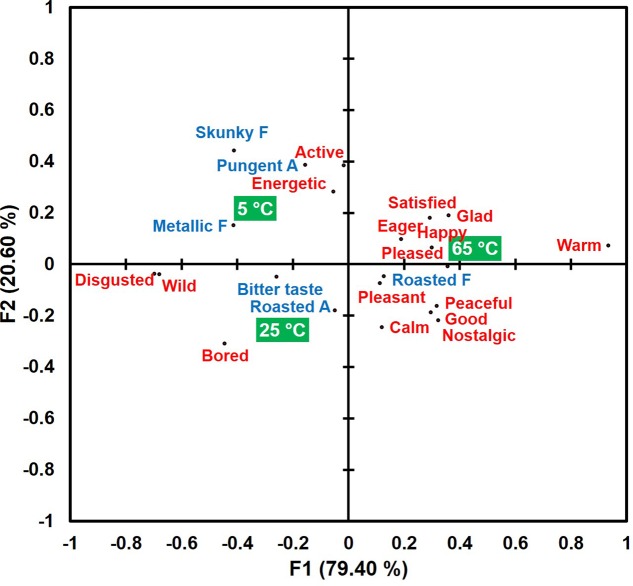
A bi-plot drawn by the correspondence analysis in the associations of sample temperatures with emotional responses (red) and sensory attributes (blue) in coffee samples evaluated at the three temperatures (green squares). “A” and “F” next to sensory attribute term represent “aroma” and “flavor,” respectively.

A bi-plot of correspondence analysis (**Figure 1**), drawn by the above 16 emotional responses and six sensory attributes, visualizes associations of sample temperatures with emotional responses and sensory attributes. More specifically, a coffee sample tasted and evaluated at 65°C was characterized more with emotion terms, “happy,” “pleased,” “satisfied,” “warm,” as well as sensory term “roasted flavor.” A coffee sample evaluated at 25°C was characterized more by emotion terms “bored” and “wild,” and by sensory attribute terms “roasted aroma” and “bitter taste.” Finally, a coffee sample consumed at 5°C was characterized more with not only sensory attribute terms “pungent aroma,” “metallic flavor,” and “skunky flavor,” but also by emotion terms of “active,” “disgusted,” and “energetic.” These results support the research propositions that specific sensory attributes (Research proposition 1) or emotional responses (Research proposition 2) can be variously dominant at hot, ambient, or cold temperature of coffee samples.

#### Gender Comparison with Respect to the Effects of Sample Temperatures on Emotional Responses and Sensory Attributes

To determine whether the proportions of participant selection for all terms of either the emotion CATA question or the sensory CATA question differed as a function of gender, the data were collapsed into two groups: females and males. Chi-square testing revealed that the proportions of selection by participants for all emotion terms were not significantly different between female (14.4%) and male (14.9%) participants (*P* = 0.51). In addition, the proportions of selection for all sensory terms were not significantly different between female (18.9%) and male (18.1%) participants (*P* = 0.28).

Cochran’s *Q*-test revealed that sample temperatures significantly affected six emotional responses (“disgusted,” “happy,” “pleased,” “satisfied,” “warm,” and “wild”) and one sensory attribute (“roasted flavor”) of brewed coffee samples from both female and male participants. However, the effects of sample temperatures on emotional responses to, and sensory attributes of, coffee samples were found to be different for 10 emotions and six sensory attributes. More specifically, for female participants, but not male participants, sample temperatures were found to affect seven emotional responses (“active,” “bored,” “calm,” “glad,” “good,” “mild,” and “peaceful”) and three sensory attributes (“skunky aroma,” “skunky flavor,” and “bitter taste”) of coffee samples. In contrast, for male participants, but not female participants, sample temperatures were found to influence three emotional responses (“nostalgic,” “pleasant,” and “worried”) and three sensory attributes (“burnt aroma,” “sour taste,” and “viscous”) of coffee samples. These results support the research proposition that the effects of sample temperatures on sensory attributes and emotional responses vary with gender (Research proposition 3).

#### Impacts of Emotional Responses and Sensory Attributes on Liking of Coffee Samples as a Function of Sample Temperature and Gender

A three-way ANOVA, treating “sample temperature” and “gender” as main effects and “participant” as a random effect, revealed that participants liked coffee samples evaluated at 65°C (mean ± SD = 6.0 ± 1.9) more than those evaluated at 25°C (4.2 ± 2.0) or 5°C (4.0 ± 2.3) (*P* < 0.001, $\eta_{p}^{2}$ = 0.30). However, there was neither a significant effect related to gender (*P* = 0.83), nor interaction between sample temperature and gender (*P* = 0.70).

**FIGURE 2:**
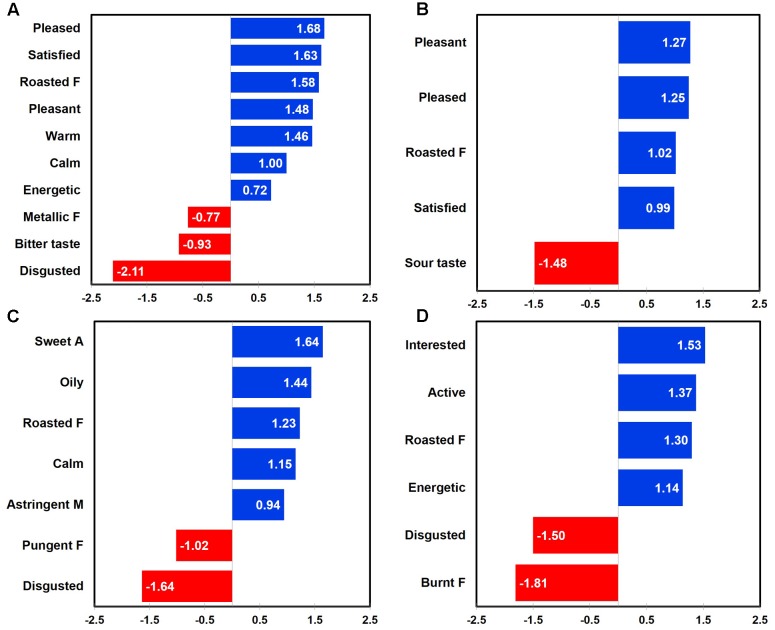
Mean drops in overall liking with respect to emotional responses and sensory attributes in coffee samples as a function of sample temperature: all temperatures **(A)**, 65°C **(B)**, 25°C **(C)**, and 5°C **(D)**. “A,” “F,” and “M” next to sensory attribute term represent “aroma,” “flavor,” and “mouthfeel,” respectively. Numerical value of each emotion or sensory attribute term represents a mean difference in overall liking between the selected and unselected cases; a positive (or negative) value for each term indicates an increase (or decrease) of overall liking between the selected and unselected cases.

Penalty-lift analysis identified drivers of liking with respect to emotional responses and sensory attributes at three different coffee sample temperatures. Overall, when considering all coffee samples tasted at three different temperatures, “pleased,” “satisfied,” “pleasant,” “warm,” “calm,” and “energetic” emotions, as well as “roasted flavor” attribute were identified as positive drivers of liking, while “disgusted” emotion, “bitter taste,” and “metallic flavor” attributes were determined as negative drivers of liking (**Figure 2A**).

When coffee samples were consumed and evaluated at 65°C, “pleasant,” “pleased,” and “satisfied” emotions as well as “roasted flavor” attribute were identified as positive drivers of liking, while a “sour taste” attribute was determined as a negative driver of liking (**Figure 2B**). In addition, not only “calm” emotion, but also “sweet aroma,” “oily,” “roasted flavor,” and “astringent mouthfeel” attributes were determined as positive drivers of liking, while both the “disgusted” emotion and the “pungent flavor” attribute were identified as negative drivers of liking for coffee sample evaluated at 25°C (**Figure 2C**). Finally, when coffee samples were evaluated at 5°C, “interested,” “active,” and “energetic” emotions, as well as the “roasted flavor” attribute were identified as positive drivers of liking, while both the “disgusted” emotion and the “burnt flavor” attribute were determined as negative drivers (**Figure 2D**). These results support the research proposition that the impact of sensory attributes and emotional responses on liking of coffee samples varies as a function of sample temperature (Research proposition 4a).

**FIGURE 3:**
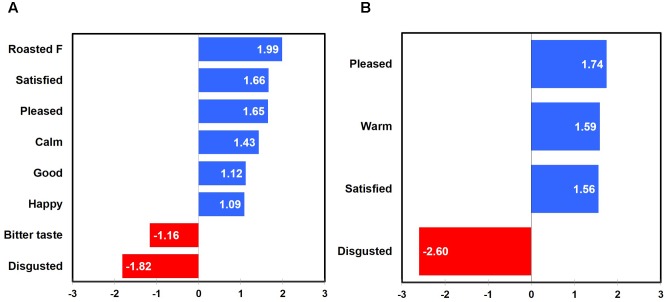
Mean drops in overall liking with respect to emotional responses and sensory attributes in coffee samples as a function of gender: females **(A)** and males **(B)**. “F” next to sensory attribute term represents “flavor.” Numerical value of each emotion or sensory attribute term represents a mean difference in overall liking between the selected and unselected cases; a positive (or negative) value for each term indicates an increase (or decrease) of overall liking between the selected and unselected cases.

Gender was found to differ with respect to positive and negative drivers of liking for coffee samples tasted at three different temperatures. For female participants, not only “satisfied,” “pleased,” “calm,” “good,” “happy” emotions, but also the “roasted flavor” attribute was identified as positive drivers of liking, while the “disgusted” emotion and the “bitter taste” attribute were determined as negative drivers of liking (**Figure 3A**). For male participants, there were only emotion-related drivers of liking, i.e., “pleased,” “warm,” and “satisfied” emotions as positive drivers and the “disgusted” emotion as a negative driver (**Figure 3B**). These results support the research proposition that the impacts of sensory attributes and emotional responses on liking of coffee samples vary as a function of gender (Research proposition 4b).

## Study 2: Effects of Sample Temperatures on Emotional Responses, and Sensory Attributes of, Green Tea

### Materials and Methods

#### Participants

Seventy-eight green tea consumers (55 females and 23 males) ranging in age from 18 to 80 years (mean ± SD = 41 ± 17) were recruited. Through a pre-screening survey, all participants self-reported that they weekly drink one or more cups of green tea without any condiments and like green tea, i.e., higher than 5-point on a 9-point hedonic scale ranging from 1 (dislike extremely) to 9 (like extremely). In addition, participants self-rated that they like both hot beverages (mean ± SD = 7.9 ± 1.1) on a 9-point hedonic scale ranging from 1 (dislike extremely) to 9 (like extremely) and cold beverages (mean ± SD = 8.2 ± 0.9). All participants were asked to refrain from eating, drinking (except water), and cigarette smoking for 2 h prior to their participation.

#### Sample Preparation and Presentation

For green tea samples, green tea bags (Korean Organic Green Tea, Nokchawon Co. Ltd., Seoul, Korea) were steeped with boiled water in a proportion of two bags per 200-mL of spring water for 3 min. For a warm-up sample, another green tea product (Sun Nokcha, Haioreum, Lyndhurst, NJ, United States) was steeped in the same manner. After steeping, the green tea was poured into a 3,000-mL stainless steel dispenser (Bunn, Springfield, IL, United States) to maintain its high temperature. Green tea samples were randomly presented at three different temperatures: 65, 25, and 5°C in a monadic sequential fashion. As for the coffee samples, sample preparation to temperatures of 25 and 5°C required green tea samples to be placed in a water bath to facilitate the cooling process. Each green tea sample (55-mL) was presented in a 118-mL white Styrofoam cup identified with a three-digit code.

#### CATA Questions of Emotion and Sensory Tests for Green Tea

An emotion CATA question, including 39 emotion terms of the EsSense Profile^®^ (King and Meiselman, 2010), was used for measuring emotional responses evoked by drinking green tea samples presented at the three serving temperatures. In addition, a sensory CATA question of green tea included 57 sensory attribute terms, based not only on previous research regarding descriptive sensory analyses of green tea (Ikeda et al., 2004; Ito and Kubota, 2005; Lee and Chambers, 2007; Lee et al., 2008; Castiglioni et al., 2015), but also on descriptions by consumer and descriptive panelists. The following attributes were included: 21 aroma attributes (animalic, ashy, beany, bitter/tannic, burnt, chemical, citrus, earthy/dirty, fermented, floral, fruity, grainy, grassy/cut grass, hay-like, herbal/herb-like, long lasting, metallic, mild/mellow, nutty, roasted, and pungent); six appearance attributes (brown color, clear, green color, sediment, turbid, and yellow color); 23 taste/flavor attributes (animalic, ashy, beany, burnt, chemical, citrus, earthy/dirty, fermented, floral, fruity, grainy, grassy/cut grass, hay-like, herbal/herb-like, long lasting, mild/mellow, nutty, roasted, pungent, bitter taste, salty taste, sour taste, and sweet taste); five mouthfeel attributes (astringent, metallic, mouth coating, smooth, and viscous); and two aftertaste attributes (bitter aftertaste and sour aftertaste).

#### Procedure

Both emotion and sensory tests of green tea samples were conducted in the same manner as described in Study 1 of coffee samples.

#### Statistical Analysis

Data was analyzed in the same manner as described in Study 1 of coffee samples.

### Results

#### Overall Effects of Sample Temperatures on Emotional Responses and Sensory Attributes

To determine whether the proportions of selection by participants for all terms of either the emotion CATA question or the sensory CATA question differed as a function of sample temperature, the data were collapsed into the three temperature conditions: 5, 25, and 65°C. Chi-square testing revealed that the proportions of selection by participants for all emotion terms significantly differed among green tea samples evaluated at the three temperatures (χ^2^ = 27.81, *P* < 0.001, *V* = 0.06): 5°C (12.8%), 25°C (12.7%), and 65°C (16.8%). More specifically, participants selected a greater number of emotion terms when evaluating green tea at 65°C than at 5 or 25°C, but the effect size (Cramér’s *V* value) was low. In addition, the proportions of selection for all sensory terms did not significantly differ among green tea samples evaluated at the three temperatures (*P* = 0.90): 5°C (17.1%), 25°C (17.1%), and 65°C (17.4%).

**Table 3** is a contingency table showing the proportions of participant selection for individual emotion terms of green tea samples tasted and evaluated at 5, 25, and 65°C. Cochran’s *Q*-test revealed that 19 emotion terms of green tea samples significantly differed as a function of sample temperature: “active,” “adventurous,” “affectionate,” “bored,” “calm,” “darling,” “disgusted,” “energetic,” “good,” “joyful,” “loving,” “nostalgic,” “peaceful,” “pleasant,” “polite,” “satisfied,” “secure,” “warm,” and “wild.” In addition, **Table 4** is a contingency table that shows the proportions of selection for individual sensory terms of green tea samples evaluated at the three temperatures. Cochran’s *Q*-test revealed that 18 sensory attributes of green tea samples significantly differed with respect to sample temperature: “animalic aroma,” “floral aroma,” “herbal/herb-like aroma,” “roasted aroma,” “pungent aroma,” “brown color,” “green color,” “yellow color,” “mild/mellow flavor,” “nutty flavor,” “roasted flavor,” “pungent flavor,” “bitter taste,” “sour taste,” “sweet taste,” “astringent mouthfeel,” “smooth mouthfeel,” and “bitter aftertaste.”

**Table 3:** A contingency table of the proportions of selection by 78 participants for individual emotion terms among green tea samples evaluated at the three different temperatures.

Terms^1^	Sample temperatures			*Q*-value	*P*-value	Cramér’s *V* value
	5°C	25°C	65°C			
Active	0.32a	0.15b	0.12b	11.42	0.003	0.22
Adventurous	0.22a	0.09b	0.03b	15.22	<0.001	0.25
Affectionate	0.01b	0.05b	0.15a	12.93	0.001	0.23
Bored	0.08b	0.21a	0.05b	10.78	0.005	0.21
Calm	0.22b	0.30ab	0.44a	8.75	0.01	0.19
Daring	0.22a	0.08b	0.08b	11.52	0.003	0.20
Disgusted	0.21a	0.24a	0.03b	19.00	<0.001	0.26
Energetic	0.35a	0.12b	0.12b	18.00	<0.001	0.28
Good	0.17b	0.22ab	0.33a	9.17	0.01	0.16
Joyful	0.18a	0.05b	0.15ab	7.30	0.03	0.17
Loving	0.01b	0.03b	0.14a	15.17	<0.001	0.24
Nostalgic	0.06b	0.03b	0.19a	12.64	0.002	0.24
Peaceful	0.14b	0.22ab	0.32a	7.59	0.02	0.18
Pleasant	0.17b	0.14b	0.33a	11.71	0.003	0.21
Polite	0.08b	0.21a	0.13ab	6.91	0.03	0.15
Satisfied	0.14b	0.14b	0.35a	14.63	<0.001	0.24
Secure	0.08b	0.10b	0.24a	12.25	0.002	0.21
Warm	0.03b	0.09b	0.63a	78.39	<0.001	0.63
Wild	0.15a	0.12ab	0.03b	9.88	0.005	0.18

*Cochran’s Q-test (Cochran, 1950), using the exact probability and distribution of the Q statistic (Patil, 1975), was performed to determine whether the proportions of participant selection for individual terms of emotion check-all-that-apply (CATA) question could differ by sample temperature. The proportions with different letters within each row represent a significant difference determined by post hoc multiple pairwise comparisons using the Marascuilo procedure (Marascuilo and McSweeney, 1967). Cramér’s V value was used to measure strength of association between two nominal variables (sample temperature × selected/unselected case) for the contingency table. Cramér’s V values, ranging from 0 (no association between the variables) to 1 (perfect association), of 0.1, 0.3, and 0.5 were considered small, medium, and large associations, respectively (Cohen, 1988). ^1^Only significant terms, determined by Cochran’s Q-test, among 39 emotion terms of the EsSense Profile^®^ (King and Meiselman, 2010) were shown (P < 0.05).*

**Table 4:** A contingency table of the proportions of selection by 78 participants for individual sensory attribute terms among green tea samples evaluated at the three different temperatures.

Terms^1^	Sample temperatures			*Q*-value	*P*-value	Cramér’s *V* value
	5°C	25°C	65°C			
**Aroma**						
Animalic	0.15a	0.00b	0.01b	22.17	<0.001	0.30
Floral	0.12b	0.13b	0.27a	9.17	0.01	0.19
Herbal/herb-like	0.26b	0.36ab	0.50a	12.70	0.002	0.21
Roasted	0.01b	0.06b	0.21a	18.10	<0.001	0.28
Pungent	0.14a	0.04b	0.01b	12.00	0.002	0.23
**Appearance**						
Brown	0.12b	0.30b	0.83a	84.93	<0.001	0.62
Green	0.26a	0.06b	0.01b	26.17	<0.001	0.33
Yellow	0.80a	0.78a	0.19b	72.10	<0.001	0.57
**Taste/flavor**						
Mild/mellow	0.09b	0.23ab	0.35a	15.05	<0.001	0.25
Nutty	0.01a	0.08ab	0.12a	7.54	0.03	0.17
Roasted	0.01b	0.09ab	0.14a	10.13	0.005	0.19
Pungent	0.21a	0.12ab	0.04b	11.04	0.004	0.21
Bitter taste	0.78a	0.78a	0.55b	13.79	0.001	0.24
Sour taste	0.18a	0.05b	0.12ab	7.50	0.03	0.16
Sweet taste	0.05b	0.12ab	0.21a	9.33	0.008	0.19
**Mouthfeel**						
Astringent	0.32a	0.28ab	0.15b	8.69	0.01	0.16
Smooth	0.31ab	0.28b	0.49a	8.60	0.01	0.18
**Aftertaste**						
Bitter aftertaste	0.95a	0.82ab	0.73b	13.27	0.001	0.24

*Cochran’s Q-test (Cochran, 1950), using the exact probability and distribution of the Q statistic (Patil, 1975), was performed to determine whether the proportions of participant selection for individual terms of sensory check-all-that-apply (CATA) question could differ by sample temperature. The proportions with different letters within each row represent a significant difference determined by post hoc multiple pairwise comparisons using the Marascuilo procedure (Marascuilo and McSweeney, 1967). Cramér’s V value was used to measure strength of association between two nominal variables (sample temperature × selected/unselected case) for the contingency table. Cramér’s V values, ranging from 0 (no association between the variables) to 1 (perfect association), of 0.1, 0.3, and 0.5 were considered small, medium, and large associations, respectively (Cohen, 1988). ^1^Only significant terms, determined by Cochran’s Q-test, among 57 sensory attribute terms were shown (P < 0.05).*

**FIGURE 4:**
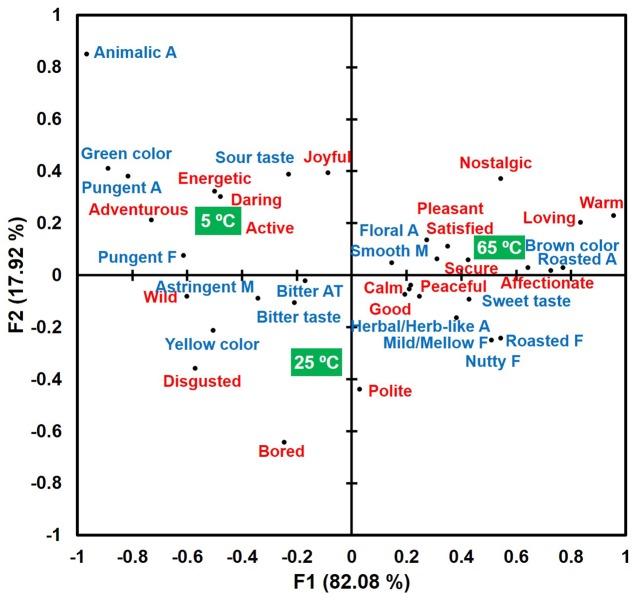
A bi-plot drawn by the correspondence analysis in the associations of sample temperatures with emotional responses (red) and sensory attributes (blue) in green tea samples evaluated at the three temperatures (green squares). “A,” “F,” “M,” and “AT” next to sensory attribute term represent “aroma,” “flavor,” “mouthfeel,” and “aftertaste,” respectively.

A bi-plot of correspondence analysis (**Figure 4**), drawn by the above 19 emotional responses and 18 sensory attributes, visualizes associations of sample temperatures with emotional responses and sensory attributes. More specifically, green tea sample tasted and evaluated at 65°C was more characterized with emotion terms “affectionate,” “calm,” “good,” “loving,” “nostalgic,” “peaceful,” “pleasant,” “satisfied,” “secure,” and “warm,” as well as sensory terms “floral aroma,” “herbal/herb-like aroma,” “roasted aroma,” “brown color,” “mild/mellow flavor,” “roasted flavor,” and “sweet taste.” Green tea samples evaluated at 25°C were more characterized by emotion terms “bored,” “disgusted,” and “polite” and the sensory term “bitter taste.” Finally, green tea samples evaluated at 5°C were characterized by emotion terms “active,” “adventurous,” “energetic,” “joyful,” and “wild,” as well as sensory terms “animalic aroma,” “pungent aroma,” “green color,” “pungent flavor,” “sour taste,” “astringent mouthfeel,” and “bitter aftertaste.” These results support the research propositions that certain sensory attributes (Research proposition 1) or emotional responses (Research proposition 2) can be more dominant at hot, ambient, or cold temperature of green tea samples.

#### Gender Comparison with Respect to the Effects of Sample Temperatures on Emotional Responses and Sensory Attributes

To determine whether the proportions of participant selection for all terms of either the emotion CATA question or the sensory CATA question differed as a function of gender, the data were collapsed into two groups: females and males. Chi-square testing revealed that the proportions of selection by participants for all emotion terms were not significantly different between female (13.7%) and male (15.1%) participants (*P* = 0.07). The proportions of selection for all sensory terms were also not significantly different between female (16.8%) and male (18.0%) participants (*P* = 0.12).

Cochran’s *Q*-test revealed that sample temperatures significantly affected two emotional responses (“disgusted” and “warm”) and five sensory attributes (“animalic aroma,” “roasted aroma,” “brown color,” “green color,” and “yellow color”) of green tea samples for both female and male participants. The effects of sample temperatures on emotional attributes and sensory attributes of green tea samples were found to differ with gender for 17 emotions and eight sensory attributes. More specifically, for female participants, but not male participants, sample temperatures were found to affect 16 emotional responses (“active,” “adventurous,” “affectionate,” “bored,” “calm,” “daring,” “energetic,” “good,” “loving,” “nostalgic,” “peaceful,” “pleasant,” “satisfied,” “secure,” “tame,” and “whole”) and seven sensory attributes (“herbal/herb-like aroma,” “beany flavor,” “mild/mellow flavor,” “roasted flavor,” “bitter taste,” “smooth mouthfeel,” and “bitter aftertaste”) of green tea samples. In contrast, for male participants, but not female participants, sample temperatures were found to influence one emotional response (“understanding”) and one sensory attribute (“sweet taste”) for green tea samples. These results support the research proposition that the effects of sample temperatures on sensory attributes and emotional responses vary with gender (Research proposition 3).

#### Impacts of Emotional Responses and Sensory Attributes on Liking of Green Tea as a Function of Sample Temperature and Gender

A three-way ANOVA, treating “sample temperature” and “gender” as main effects and “participant” as a random effect, revealed that hedonic ratings of green tea samples differed significantly with respect to sample temperature (*P* < 0.001, $\eta_{p}^{2}$ = 0.22): at 65°C (mean ± SD = 6.3 ± 1.7) > at 25°C (5.3 ± 1.8) > at 5°C (4.6 ± 2.1). However, there were no significant effects of gender (*P* = 0.33), or interaction between sample temperature and gender (*P* = 0.28).

**FIGURE 5:**
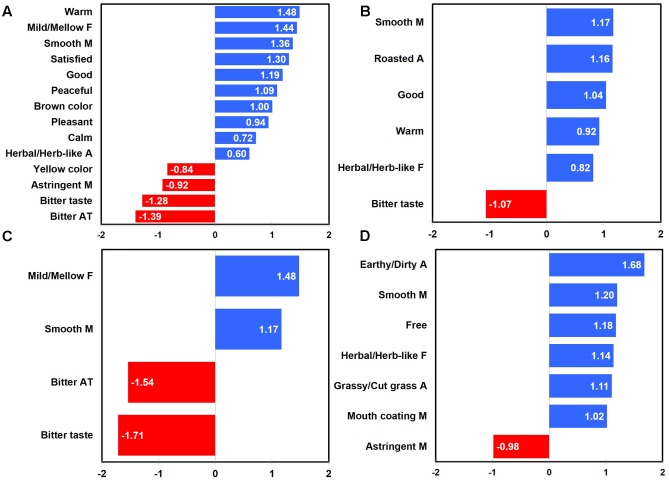
Mean drops in overall liking with respect to emotional responses and sensory attributes in green tea samples as a function of sample temperature: all temperatures **(A)**, 65°C **(B)**, 25°C **(C)**, and 5°C **(D)**. “A,” “F,” “M,” and “AT” next to sensory attribute term represent “aroma,” “flavor,” “mouthfeel,” and “aftertaste,” respectively. Numerical value of each emotion or sensory attribute term represents a mean difference in overall liking between the selected and unselected cases; a positive (or negative) value for each term indicates an increase (or decrease) of overall liking between the selected and unselected cases.

Penalty-lift analysis identified drivers of liking with respect to emotional responses and sensory attributes at three different temperatures of green tea sample. Overall, when considering all green tea samples experienced at three different temperatures, “warm,” “satisfied,” “good,” “peaceful,” “pleasant,” and “calm” emotions, as well as “mild/mellow flavor,” “smooth mouthfeel,” and “brown color” attributes were identified as positive drivers of liking. Additionally, “bitter aftertaste,” “bitter taste,” “astringent mouthfeel,” and “yellow color” attributes were identified as negative drivers of liking for green tea samples evaluated at different temperatures (**Figure 5A**).

Positive and negative drivers of liking with respect to emotional responses and sensory attributes were found at three different temperatures of green tea sample. When green tea samples were consumed and evaluated at 65°C, not only “good” and “warm” emotions, but also “smooth mouthfeel,” “roasted aroma,” and “herbal/herb-like flavor” attributes were identified as positive drivers of liking, while the “bitter taste” attribute was determined as a negative driver of liking (**Figure 5B**). When green tea samples were evaluated at 25°C, only sensory attributes were identified as positive and negative drivers of liking: i.e., “mild/mellow flavor” and “smooth mouthfeel” attributes as positive drivers and “bitter taste” and “bitter aftertaste” as negative drivers of liking (**Figure 5C**). In addition, when green tea samples were evaluated at 5°C, not only the “free” emotion, but also “earthy aroma,” “smooth mouthfeel,” “herbal/herb-like flavor,” “grass/cut grass aroma,” and “mouth coating” attributes were identified as positive drivers of liking, while the “astringent mouthfeel” attribute was determined as a negative driver of liking (**Figure 5D**). These results support the research proposition that the impacts of sensory attributes and emotional responses on liking of green tea samples vary as a function of sample temperature (Research proposition 4a).

**FIGURE 6:**
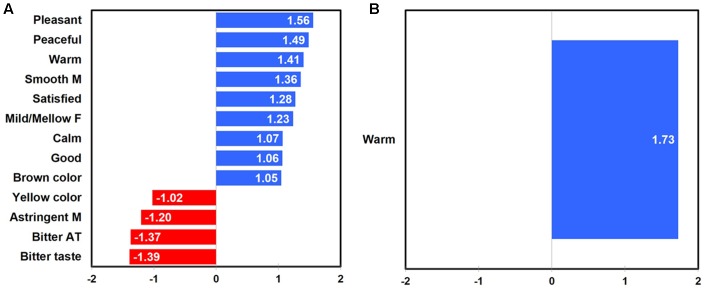
Mean drops in overall liking with respect to emotional responses and sensory attributes in green tea samples as a function of gender: females **(A)** and males **(B)**. “F,” “M,” and “AT” next to sensory attribute term represent “flavor,” “mouthfeel,” and “aftertaste,” respectively. Numerical value of each emotion or sensory attribute term represents a mean difference in overall liking between the selected and unselected cases; a positive (or negative) value for each term indicates an increase (or decrease) of overall liking between the selected and unselected cases.

Positive and negative drivers of liking for green tea samples tasted at three different temperatures were found to differ between female and male participants. For female participants, “pleasant,” “peaceful,” “warm,” “satisfied,” “calm,” and “good” emotions as well as “smooth mouthfeel,” “mild/mellow flavor,” and “brown color” attributes were identified as positive drivers of liking. In addition, four sensory attributes, i.e., “bitter taste,” “bitter aftertaste,” “astringent mouthfeel,” and “yellow color,” were determined as negative drivers of liking (**Figure 6A**). For male participants, only the “warm” emotion was identified as a positive driver of liking for green tea samples evaluated at different temperatures (**Figure 6B**). These results support the research proposition that the impacts of sensory attributes and emotional responses on liking of green tea samples vary as a function of gender (Research proposition 4b).

## Discussion

### Variations with Respect to Emotional Responses and Sensory Attributes as a Function of Sample Temperature of Coffee and Green Tea (Research Propositions 1 and 2)

In most sensory studies of hot foods or beverages, samples have been evaluated in a temperature range at which those samples are typically consumed. For example, brewed coffee has been evaluated at one specific temperature between 75 and 55°C (Nebesny and Budryn, 2006; Seo et al., 2009a,b; Bhumiratana et al., 2014; Di Donfrancesco et al., 2014). However, it is well-known that people consume hot foods or beverages over a wider range of temperature in everyday life, and certain beverages such as coffee and tea are often consumed at both hot and cold temperatures. Nevertheless, little attention has been paid to whether and how emotional responses as well as sensory attributes of hot foods or beverages can change as a function of their product temperatures.

The results from this study showed the dynamics of sensory attributes in both coffee and green tea samples with respect to serving temperatures of 65, 25, and 5°C. Six and 18 sensory attributes of coffee and green tea samples, respectively, significantly differed in terms of sample temperature. Sample temperature-induced changes in sensory attributes of brewed coffee have been also observed in other studies (Stokes et al., 2016; Steen et al., 2017). In a recent study conducted by Stokes et al. (2016), “coffee flavor,” “roasted/burnt flavor,” and “full body” attributes were more associated with brewed coffee samples evaluated at higher temperatures of 60.4, 70.8, and 74.4°C, while “earthy flavor” and “sour/acidic taste” were more related to those evaluated at lower temperatures of 31.0 and 41.1°C. The present study to some extent showed similar results, that “roasted flavor” attribute of brewed coffee was more often identified at higher temperature (65°C), while “pungent aroma,” “metallic flavor,” and “skunky flavor” attributes were more often characterized at lower temperature (5°C). To the authors’ best knowledge, this study was the first to demonstrate that sensory attributes of green tea can vary with sample temperatures. Notably, green tea samples showed a greater number of significant sensory attributes affected by sample temperatures than did coffee samples, indicating that sensory attributes of green tea samples were more sensitive to temperature changes than those of coffee samples. Like coffee samples, green tea samples were more frequently characterized using desirable sensory attributes at higher temperature (65°C), while those at lower temperatures (5 and 25°C) were more often described using undesirable attributes.

Both coffee and green tea samples evaluated at higher temperature (65°C) were more often characterized using emotions of positive valence with either high or low level of activation/arousal. In other words, beverage samples consumed at 65°C more frequently evoked emotions of positive valence, such as “pleased,” “happy,” “satisfied,” and “warm,” etc., than did those consumed at either 25 or 5°C. In addition, while beverage samples consumed and evaluated at 25°C were characterized with emotions of negative valence with low level of activation/arousal, those evaluated at 5°C were described with emotions of negative valence with high level of activation/arousal. Sample temperature-induced variation with respect to emotional responses might be associated with the dynamics of sensory attributes among the three temperature conditions of coffee or green tea samples. More specifically, the tetrachoric correlation analysis (Divgi, 1979) of sensory attributes and emotional responses for coffee samples revealed that “roasted flavor” and “mouth-coating” attributes showed positive correlation with emotions of positive valence, such as “warm” (roasted flavor: +0.54, mouth-coating: +0.34), “pleasant” (+0.49, +0.18), “satisfied” (+0.37, +0.20), “pleased” (+0.32, +0.22), and “happy” (+0.22, +0.13). In addition, “pungent aroma,” “chemical flavor,” “metallic flavor,” and “bitter taste” attributes positively correlated with emotions of high level of activation/arousal, such as “active” (pungent aroma: +0.08, chemical flavor: +0.18, metallic flavor: +0.17, and bitter taste: +0.22), “eager” (+0.14, +0.08, +0.21, and +0.09), and “energetic” (+0.28, +0.22, +0.18, and +0.15). These results were in agreement with previous studies that showed associations between sensory attributes and emotional responses (Seo et al., 2009c; Porcherot et al., 2010; Chaya et al., 2015). Furthermore, the tendency of warmer food or beverage products to evoke positive emotions illustrated the “temperature-premium effect,” where exposure to warm temperatures can increase a consumer’s evaluation of a product through the activation of the concept of positive emotional warmth in an individual, leading to greater positive reactions (Zwebner et al., 2013). Such an affective response of thermal stimuli could be explained by the increased neural activations in the brain regions associated with thermal sensation, sensory discrimination, emotional awareness and processing, and cognitive processing during direct exposure to warm stimulation (Sung et al., 2007).

### Gender Effects on the Sample Temperature-Induced Variations with Respect to Emotional Responses to, and Sensory Attributes of, Coffee and Green Tea (Research Proposition 3)

Influences of sample temperatures on emotional responses and sensory attributes were observed in both female and male participants. Even though female and male participants did not exhibit any significant differences in terms of the proportions of selection by participants for either all emotion terms or all sensory terms, the results for female participants showed a larger number of emotion and sensory terms that significantly varied as a function of sample temperature compared to those of male participants. In other words, female participants displayed more consensus and less variable responses toward coffee and green tea samples presented at three different temperatures. This result might be related to earlier findings that female participants outperformed male participants with respect to odor sensitivity, odor identification, odor memory, and verbal proficiency (Doty et al., 1984; Larsson et al., 2003; Doty and Cameron, 2009; Ferdenzi and Roberts, 2013) although gender differences were not always observed. Moreover, a functional neuroimaging study conducted by Royet et al. (2003) found that while males showed neural activations in their bilateral insula and left piriform–amygdala regions during hedonic judgment of odors, females showed neural activations not only in the same regions as male participants, but also in left orbitofrontal cortex related to odor identification, language, and emotion. Females have also been found to be more emotionally expressive toward foods and beverages than males (King et al., 2010; Jaeger and Hedderley, 2013).

### Impacts of Emotional Responses and Sensory Attributes on Likings of Coffee and Green Tea (Research Proposition 4)

Our findings support previous research suggesting that not only sensory attributes, but also emotional responses to some extent contribute to overall liking of foods and beverages (Seo et al., 2009c; Samant et al., 2017). Interestingly, drivers of liking with respect to emotional responses and sensory attributes were found to differ as a function of sample temperature in both coffee and green tea samples. For coffee samples, only the “roasted flavor” attribute was observed as a positive driver of liking at all three temperatures, while positive and negative drivers of liking changed at each temperature. “Roasted flavor” was more often identified at higher temperature (65°C), possibly suggesting that participants increasingly like brewed coffee served at 65°C the most. For green tea, the “smooth mouthfeel” attribute served as a positive driver of liking at all three temperatures. Like coffee samples, positive and negative drivers of liking for green tea samples varied with sample temperatures. Overall, among 57 sensory attributes of green tea samples, “mild/mellow flavor,” “smooth mouthfeel,” “brown color,” and “herbal/herb-like aroma” were found to be positive drivers of liking, while “bitter aftertaste,” “bitter taste,” “astringent mouthfeel,” and “yellow color” were negative drivers of liking. This result was in agreement with previous research where the United States consumers liked green tea samples with “mild flavor,” “no aftertaste,” “weak bitterness,” “flowery or fruity flavor,” and “brown flavor” notes (Lee et al., 2010). In addition, “sweet taste” and “roasted-related flavors” were considered to be drivers of liking for green tea samples (Lee et al., 2008). Building on previous research regarding sensory drivers of liking for green tea samples, this study added empirical evidence that emotions also serve as drivers of liking for green tea samples. Specifically, “warm,” “satisfied,” “good,” “peaceful,” “pleasant,” and “calm” emotions were found to play important roles in modulating liking of green tea samples served at different temperatures.

It is worth noting that drivers of liking for coffee or green tea samples were found to differ between female and male participants. While both emotional responses and sensory attributes contributed to likings of beverage samples among female participants, only emotional responses were considered as drivers of liking among male participants. This result might be related to previous findings that females outperformed males in odor sensitivity, odor identification, and odor memory tasks (Doty et al., 1984; Larsson et al., 2003; Doty and Cameron, 2009; Ferdenzi and Roberts, 2013). Females have also been found to perform better in taste sensitivity tasks than males (Michon et al., 2009). Since females could better detect sample temperature-induced changes in sensory attributes than males, sensory attributes might contribute to likings of coffee and green tea samples among female participants, but not among male participants.

## Conclusion

To summarize, the results of this study showed that both emotional responses to, and sensory attributes of, coffee or green tea samples can vary with sample temperature. In other words, people may experience different sensory attributes and emotions with decreasing temperature of brewed coffee or green tea beverages, affecting their likings of those beverages. In addition, sample temperature-induced variations with respect to emotional responses and sensory attributes differed between female and male participants. Furthermore, while sensory attributes as well as emotional responses were found to be drivers of liking among female participants, only emotional responses were identified as drivers of liking among male participants. In conclusion, our findings provide empirical evidence that emotional responses to, and sensory attributes of, coffee and green tea beverages can vary as a function of sample temperature, and that such temperature-induced variations can differ by gender. Our findings emphasize the need to consider product temperature-induced dynamics of emotional responses and sensory attributes when evaluating food or beverage products that are temperature-sensitive. In other words, processors, manufacturers, sensory professionals, and marketers in the food industry should put more effort into exploring emotional responses to, and sensory attributes of, food or beverage products over the wider range of product temperatures that consumers may encounter in daily life. Such efforts may lead to both a better understanding of product characteristics and increases in consumer acceptance and purchase intent.

## Author Contributions

RP and H-SS conceived and designed the study. RP collected the data, and RP and H-SS analyzed the data. RP and H-SS participated manuscript preparation and approved the final manuscript.

## Conflict of Interest Statement

The authors declare that the research was conducted in the absence of any commercial or financial relationships that could be construed as a potential conflict of interest.
